# Identification of a molecular system that regulates growth cone membrane potential during growth cone guidance

**DOI:** 10.1186/1471-2202-12-S1-P280

**Published:** 2011-07-18

**Authors:** Tatsuya Yamada, Henri C Jimbo, Shin Ishii, Makoto Nishiyama, Kyonsoo Hong, Yuichi Sakumura

**Affiliations:** 1 Grad. School of Information Science, Nara Institute of Science and Technology, Ikoma, Nara, 630-0192, Japan; 2 Grad. School of Biological Science, Nara Institute of Science and Technology, Ikoma, Nara, 630-0192, Japan; 3 Grad. School of Informatics, Kyoto University, Uji, Kyoto, 611-0011, Japan; 4 Department of Biochemistry, New York University School of Medicine, New York, NY 10016, USA

## 

Growth cones, at the tips of growing neurites (axons and dendrites) of developing neurons are guided to their synaptic targets by various external guidance molecules to make functional neuronal connections. The mechanisms by which growth cones respond to these external guidance molecules are yet to be elucidated. In cultured *Xenopus* spinal commissural interneurons (CINs) derived from stage 26-28 embryos, the magnitude of cyclic-GMP (cGMP) signaling determines the direction of growth cone turning in response to a secreted guidance molecule, semaphorin 3A (Sema3A) by controlling growth cone membrane potential shifts [1]. The molecular mechanism of intracellular signaling that controls the growth cone membrane potential is largely unknown. In this study, we aim to identify the molecular interaction between the signaling cascade(s) that regulate(s) the activities of Na^+^ channels (NaC) and Cl^–^ channels (ClC) by employing Bayesian statistics with parametric models for the computational analysis of experimentally derived data.

**Figure 1:**
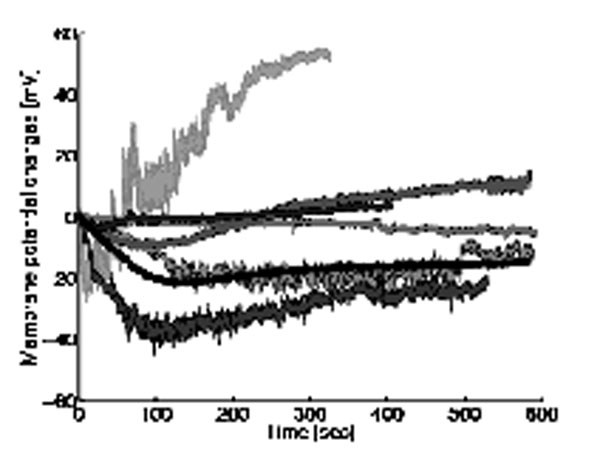
Time courses of membrane potential shifts in control condition, showing the variability in cellular response to the identical cGMP stimulus (10 μM cGMP).

Measurements of membrane potential in cultured *Xenopus* spinal CIN growth cones were performed in the absence (control) and presence of the following pharmacological drugs: DNDS (ClC blocker), STX (NaC blocker) and KT5823 (PKG inhibitor) (Fig. 1). The following conjectures were applied in the model: 1) Both NaC and ClC are regulators of growth cone membrane potential shifts in the control. 2) In the presence of either DNDS or STX, one of the ionic channels does not function without changing the intracellular molecular system. 3) In the presence of KT5823, the molecular system is modified by inhibition of the PKG-mediated signaling cascade, which is required for activation of the NaC. Under these assumptions, if an interaction occurs between the cascades that regulate NaC and ClC, the time courses of membrane potential shifts in the presence of STX should be different from those in the presence of KT5823. However, a problem exists in modeling because of the variability in the cellular response to a given cGMP stimulus (Fig. 1) that maybe due to various cellular characteristics, e.g., growth cone shape, channel distribution, and developmental stage of the animal. Thus, using a statistical characterization of the molecular system, we examined the existence of such an interaction based on the ability to reconstruct the time courses in the control condition.

We reconstructed time courses of membrane potential shifts from the data set of DNDS/STX or DNDS/KT5823. We introduced the Hill-type formula as a parametric expression of the time course since most feedforward biochemical signals show a monotonic increase. The cellular variability in this framework can be modeled as the probability distributions of the parameters, which is approximated with the Gamma distributions. We estimated the optimized hyper-parameters in the Gamma distributions for all pharmacological conditions in which the posterior distributions are maximized. The difference in the hyper-parameters between the STX condition and the KT5823 condition suggests that there exists an interaction between the cascades that regulate the NaC and ClC.
